# Trinity review: integrating Registered Reports with research ethics and funding reviews

**DOI:** 10.1186/s13104-022-06043-x

**Published:** 2022-05-19

**Authors:** Yuki Mori, Kaito Takashima, Kohei Ueda, Kyoshiro Sasaki, Yuki Yamada

**Affiliations:** 1 Graduate School of Human-Environment Studies, Kyushu University, 744 Motooka, Nishi-ku, Fukuoka, 819-0395 Japan; 2 Faculty of Informatics, Kansai University, 2-1-1, Ryozenji-cho, Takatsuki, Osaka, 569-1095 Japan; 3 Faculty of Arts and Science, Kyushu University, 744 Motooka, Nishi-ku, Fukuoka, 819-0395 Japan

**Keywords:** Registered Reports, Research ethics, Research grants, Peer review, Review system, Academic publishing

## Abstract

One major source of exhaustion for researchers is the redundant paperwork of three different documents—research papers, ethics review applications, and research grant applications—for the same research plan. This is a wasteful and redundant process for researchers, and it has a more direct impact on the career development of early-career researchers. Here, we propose a trinity review system based on Registered Reports that integrates scientific, ethics, and research funding reviews. In our proposed trinity review system, scientific and ethics reviews are undertaken concurrently for a research protocol before running the study. After the protocol is approved in principle through these review processes, a funding review will take place, and the researchers will begin their research. Following the experiments or surveys, the scientific review will be conducted on a completed version of the paper again, including the results and discussions (i.e., the full paper), and the full paper will be published once it has passed the second review. This paper provides the brief process of the trinity review system and discusses the need for and benefits of the proposed system. Although the trinity review system only applies to a few appropriate disciplines, it helps improve reproducibility and integrity.

## Introduction

Early-career researchers (ECRs) need to undertake productive scientific research with the aim of obtaining a degree, notwithstanding the huge chasm between the classical research practices imparted by professors and the state-of-the-art research practices required in a drastically transforming scientific ecosystem. For example, a new submission format called “Registered Reports” (RRs) has emerged, and it is reported that 77% of researchers who used RRs are ECRs, whereas only 4% are professors [1]. Extending RRs, which are widely used by ECRs whose productivity is important, in more conducive directions would help boost their research activities. Thus, we will present here a draft sketch of a new review system that we, the ECRs, believe will be more efficient, flexible, and diverse. Current researchers are required to write three different documents for each project. One is a research paper manuscript. Research papers are academic publications that describe some of the findings of a research project, and many researchers focus on producing these peer-reviewed publications. The second is an application for ethics review. In experimental psychology, for example, researchers conduct experiments on living things, including humans (i.e., subjects). To ensure that subjects’ rights and safety are not violated, the study plan is reviewed in advance by the ethical committee of affiliated institutions. This holds true for any research that involves humans or animals as subjects, such as medical research [2]. Researchers can start their studies when the plan is approved and does not pose any ethical problems [3]. Similar to RRs, the ethics review applications are peer-reviewed and often revised before studies are conducted.

The authors also often write applications for research grants to conduct their studies. Funds are necessary for research activities. Researchers submit dozens of pages outlining the plans for their studies to funding agencies to obtain funds for their research. The researchers will be awarded grants if the agencies deem the plans beneficial. The important thing here is that the grant application is also peer-reviewed and selected before the studies. A study reported that each grant proposal takes researchers an average of 34 working days [4]. If the researchers fail to obtain grants, they waste 34 working days. Obtaining research grants is a lifeline for researchers, and if they fail to obtain grants early in their research career, it will be very difficult for them to obtain grants in the middle or later stages of their careers [5].

The post-study peer review process comes too late; peer review is more helpful when it comes earlier in the process. The reviewers’ comments in post-study peer review cannot improve the manuscript as suggestions that should have been made during the research design stage are often provided after all studies have been completed. It is also necessary to move back and forth between ethics and scientific review to satisfy both positions when there are different suggestions for ethics review and scientific review. It could be difficult for many researchers. Furthermore, post-study peer review contributes to publication bias. Publication bias motivates researchers to engage in questionable research practices (QRPs), such as *p*-hacking, and consequently reduces the reproducibility of findings and integrity.

Recently, it has become relatively common to submit papers in the form of RRs [6, 7] to suppress publication bias and QRPs. RRs are an editorial system in which the authors submit a research protocol to a journal before running the study (Stage 1). During Stage 1, the value of the research question, the rationale for the hypothesis, and the validity of the methods are assessed [6]. This allows reviewers to assist authors in improving their protocol and rationale or to make changes that will further improve the quality of the paper [8]. As a result of peer review and revision, if the protocol is judged to be publishable, the protocol and results are accepted in principle for publication, regardless of the results of studies performed afterward. After the studies, a completed version of the paper is submitted again for peer review, and a final decision on publication is made (Stage 2). Despite the benefits of RRs for improving reproducibility and the integrity of science, RRs are not prevalent, even in the disciplines where RRs have already been introduced, such as psychology and life sciences [1]. Furthermore, while RRs are more actively used by ECRs, it has been pointed out that the financial cost of securing participants to satisfy statistical power is a barrier to the use of RRs, especially for researchers with limited resources [6].

As mentioned above, researchers submit similar documents to three different organizations, each with its own format, and each is independently peer-reviewed three times. The research ethics and funding reviews also assess scientific validity and its importance, which overlap with the peer review of academic papers, wasting the effort of the reviewers involved in all three. This is a wasteful and redundant process. Furthermore, post-study peer review might not only waste reviewers’ time and effort improving manuscripts but might also be a breeding ground for QRPs. While using RRs could suppress QRPs and improve reproducibility, more widespread use of RRs requires motivating researchers to use RRs and financially supporting researchers who are willing to use RRs [6]. Hence, we propose to integrate RRs with research ethics and funding reviews. A system that integrates RRs with funding reviews has already been proposed [1, 9]. By integrating a research ethics review, the proposed method significantly reduces researchers’ workloads more than the conventional system, which entails redundant writing tasks.

## Main text

In the trinity review system, researchers will submit a detailed pre-research protocol in the Stage 1 manuscript of the RRs. As in typical RRs, the protocol will be peer-reviewed by several reviewers. This involves assessing the value of the research question, the rationale for the hypothesis, and the validity of the methods for testing the hypothesis^1^ In our proposed trinity review system, an ethics review will be conducted concurrently with the Stage 1 protocol. Some reviewers dedicated to ethical aspects will be assigned to the protocol and review the ethical aspects of the paper. Protocols with ethical problems are returned to the authors, who would revise them according to the reviewer’s comments and the editor’s decision. If there is some necessity in terms of ethical considerations that are not covered in the code, the protocol is reviewed additionally by the author’s institution (e.g., in the medical field, institutional acknowledgement might be necessary if the research uses some invasive methods against humans). When the protocol is accepted through these review processes, a funding review would be initiated. Here, the Stage 1 protocols and funders would be matched in some way. The funders assess whether the Stage 1 protocols are possibly beneficial to them or the public and invest grants in protocols that meet their criteria. In this way, the trinity review system unifies the three different types of peer reviews and makes them run smoothly.

**Fig. 1:**
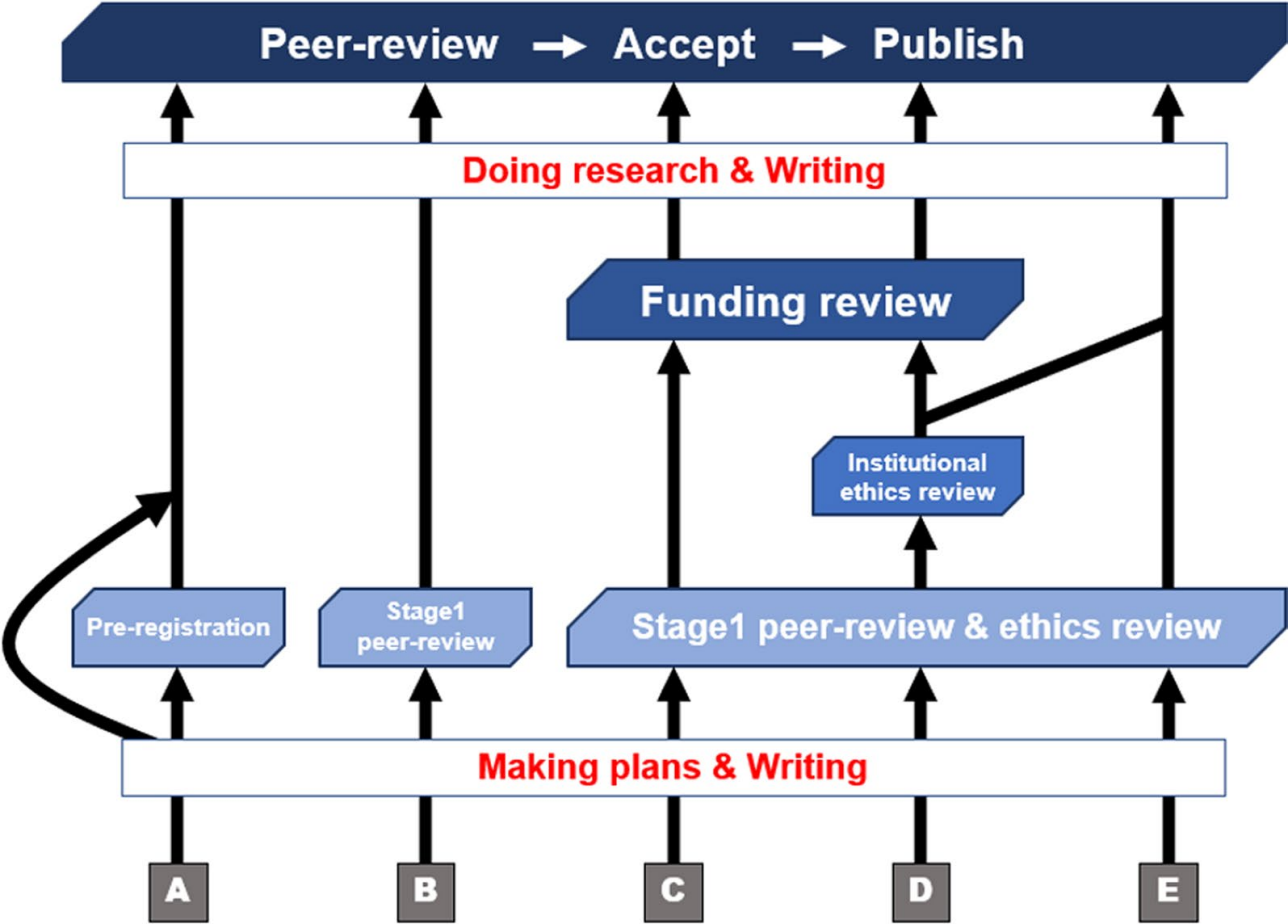
The trinity review system as one of the various publishing processes*.* This figure shows the trinity review system as one of the various publication processes and the publication process options available to researchers. **A** Traditional publishing flows: preregistration by each researcher on a voluntary basis is recommended. **B** Flow of the typical and current RRs: at Stage 1, a manuscript that describes methods, plans, hypotheses, and so on in detail is peer-reviewed. After in-principle acceptance, researchers begin the experiments or investigations. Then, the Stage 2 manuscript, including results and discussion, is peer-reviewed and published. **C** The most orthodox flow in the trinity review: an ethics review is conducted at the same time as Stage 1 peer-review for the same manuscript. After in-principle acceptance, a funding review is conducted, and researchers then start experiments or investigations. Finally, the Stage 2 manuscript, including results and discussion, is peer-reviewed and published. **D** Manuscripts on the trinity review flow, which have some special necessity, are sent to the institute to which authors belong, and are then subjected to a second ethics review. We suppose that the ethics review of the trinity review system is conducted under the code of ethics determined by each journal that approves the trinity review. However, if there is some necessity in terms of ethical considerations that are not covered in the code, the protocol is reviewed along with this flow (e.g., in the medical field, institutional acknowledgement might be necessary if the research uses some invasive methods against humans). Then, researchers start experiments or investigations, and the Stage 2 manuscript will be published after peer-review. **E** Trinity review flow without a funding review. Researchers who do not need funding for the research but want to use the Stage 1 peer-review with ethics review for the same manuscript will use this system

Funding review (or matching) is probably the trickiest part of this system. The funding review would not require any revisions to the manuscript, and thus it would only take place after the protocol has passed scientific and ethics reviews and been accepted in principle. Research funds are provided for protocols, not for individual researchers as in a typical funding review. Two ways of matching protocols and funders are possible. First, funders select and invest in the protocol they are interested in from a list of in-principle accepted protocols that can be browsed only by the funders. Second, funders offer grants; these offerings are listed, and authors apply for those that match their protocols. This is similar to the traditional grant system, but it is qualitatively different because the reviewed manuscripts ensure the importance of the research question, the relevance of the protocol, and research ethics. For both matching ways, authors do not need to write new documents for research grants because in-principle accepted protocols are used as the application form. These methods help researchers to avoid spending time on time-wasting paperwork, and funders can easily assess whether the research is truly suitable for funding. Note that this funding review only provides an opportunity or option to obtain grants. In other cases, authors could choose to skip the funding review. For example, they can cover research expenses in another way (e.g., having another grant outside the trinity review system or using crowdfunding); nevertheless, they still want to receive a combined review from scientific and ethical perspectives (Fig. 1)^2^.

There are some obvious potential difficulties in implementing the trinity review. One might argue that it will increase the workload for journals. The key point is that it requires several ethics reviewers, which could lead to a shortage of reviewers and incentives for them. Next, this trinity review is suitable for disciplines and communities that already have some common rules, guidelines, and formats for ethics reviews; otherwise, the journal will need to prepare them, which will incur additional costs. Furthermore, the trinity review system requires ethics reviewers in addition to academic reviewers. Many journals are already experiencing difficulties in finding reviewers [11]. The addition of ethics reviews could be a burden for them.

There are a number of issues that need to be resolved when introducing the trinity review system. However, this system is necessary to promote more efficient and proper research practices and to financially support researchers, especially ECRs, who work to improve the reproducibility and integrity of research. Here, we point out the benefits that the trinity review system brings to the academic community.

The first benefit is that this system eliminates the redundancy of reviewers as well as authors, and leads to more efficient research practice. As already mentioned, this system combines the Stage 1 manuscript of RRs with ethics review and funding review to eliminate the redundancy of authors preparing multiple paperwork for a single study. In addition, scientific and ethics reviews are conducted simultaneously on the same documents. This system provides academic and ethical peer review of protocols before the study is conducted so that reviewers’ comments can be used to improve the quality of protocols. This also helps to eliminate the redundancy of reviewers.

The second benefit is that this system improves reproducibility and research integrity. This system is based on RRs, and any protocols using this system will be published regardless of the statistical significance of the results. As a result, it could prevent file drawer problems [12] and publication bias [13, 14], questionable research practices or research misconduct, and, as a result, increase reproducibility [8, 15] in the same way as traditional RRs. In addition, papers published through the trinity review system will provide a detailed description of ethical considerations. In this system, an ethics review will be conducted, as well as a scientific review, at Stage 1. Consequently, researchers must describe ethical considerations in greater detail in their protocols. This will help readers determine what specific considerations have been made. Furthermore, since scientific and ethics reviews are conducted simultaneously through the same documents, the authors have no opportunity to arbitrarily modify the original plan regarding ethical considerations without consulting the journal, as per the process of traditional RRs. This will prevent the problem of changing ethical operations after the ethics assessment is completed [16], and hence problematic ethical behaviors will be suppressed.

The third benefit is that this system motivates researchers to use RRs. This system provides the option to obtain research funds. Therefore, not only resource-constrained researchers who are willing to use RRs, but also researchers with limited funding, especially ECRs, can make use of the trinity review system to promote the use of RRs. The trinity review system also benefits researchers who belong to institutes or departments that do not have an institutional review board and independent researchers who do not belong to any research institution [17, 18]. By using this system, they can have an opportunity to review their protocol from an ethical perspective quickly and easily. This can promote citizen science as well as the use of RRs.

## Outlook

In summary, the trinity review system would be valuable for making research practice more efficient. Moreover, the trinity review system is beneficial for reproducibility and research integrity because it suppresses questionable practices involving research, ethical considerations, and the use of funds. This method is easier to implement in research areas and specific research topics where the use of RRs is already popular and well established. In contrast, this system cannot be applied to exploratory and conceptual disciplines (e.g., humanities and theoretical physics) and studies that are not suitable for using RRs (e.g., developing new engineering techniques and optical illusions [19]). Thus, since the trinity review system will probably not become the standard for all research and because it is not clear whether RRs themselves will continue to be used [20], the system can only play a temporary and tentative role (Fig. 1).

Nevertheless, it would be a significant step forward in developing a system that allows researchers to concentrate on the task at hand and eliminate waste of effort and time in their daily work.

## Data Availability

Not applicable.

## Abbreviations

*ECRs*: Early career researchers
*PCI*: Peer community in
*QRPs*: Questionable research practices
*RRs*: Registered Reports

## Footnotes

1 Note that, for exploratory research RRs, it is also important to assess the validity of explorations and the transparency of its process after completing Stage 1 [10].

2 For more detailed specifics of the process and proposals for a possible trinity review system, please refer to supplemental materials (https://osf.io/rq5vb/).
